# Aortic flow patterns and wall shear stress maps by 4D-flow cardiovascular magnetic resonance in the assessment of aortic dilatation in bicuspid aortic valve disease

**DOI:** 10.1186/s12968-018-0451-1

**Published:** 2018-04-26

**Authors:** José Fernando Rodríguez-Palomares, Lydia Dux-Santoy, Andrea Guala, Raquel Kale, Giuliana Maldonado, Gisela Teixidó-Turà, Laura Galian, Marina Huguet, Filipa Valente, Laura Gutiérrez, Teresa González-Alujas, Kevin M. Johnson, Oliver Wieben, David García-Dorado, Arturo Evangelista

**Affiliations:** 1 Hospital Universitari Vall d’Hebron, Department of Cardiology. Vall d’Hebron Institut de Recerca (VHIR), Universitat Autònoma de Barcelona, Paseo Vall d’Hebron 119-129, 08035 Barcelona, Spain; 2 Cardiac Imaging Department, CETIR-ERESA, Clínica del Pilar-Sant Jordi, Barcelona, Spain; 3 Departments of Medical Physics & Radiology, University of Wisconsin – Madison, Madison, WI USA

**Keywords:** Bicuspid aortic valve, 4D flow cardiovascular magnetic resonance (4D flow CMR), Wall shear stress, Ascending aorta, Aorta hemodynamics, Aortic dilatation

## Abstract

**Background:**

In patients with bicuspid valve (BAV), ascending aorta (AAo) dilatation may be caused by altered flow patterns and wall shear stress (WSS). These differences may explain different aortic dilatation morphotypes. Using 4D-flow cardiovascular magnetic resonance (CMR), we aimed to analyze differences in flow patterns and regional axial and circumferential WSS maps between BAV phenotypes and their correlation with ascending aorta dilatation morphotype.

**Methods:**

One hundred and one BAV patients (aortic diameter ≤ 45 mm, no severe valvular disease) and 20 healthy subjects were studied by 4D-flow CMR. Peak velocity, flow jet angle, flow displacement, in-plane rotational flow (IRF) and systolic flow reversal ratio (SFRR) were assessed at different levels of the AAo. Peak-systolic axial and circumferential regional WSS maps were also estimated. Unadjusted and multivariable adjusted linear regression analyses were used to identify independent correlates of aortic root or ascending dilatation. Age, sex, valve morphotype, body surface area, flow derived variables and WSS components were included in the multivariable models.

**Results:**

The AAo was non-dilated in 24 BAV patients and dilated in 77 (root morphotype in 11 and ascending in 66). BAV phenotype was right-left (RL-) in 78 patients and right-non-coronary (RN-) in 23. Both BAV phenotypes presented different outflow jet direction and velocity profiles that matched the location of maximum systolic axial WSS. RL-BAV velocity profiles and maximum axial WSS were homogeneously distributed right-anteriorly, however, RN-BAV showed higher variable profiles with a main proximal-posterior distribution shifting anteriorly at mid-distal AAo. Compared to controls, BAV patients presented similar WSS magnitude at proximal, mid and distal AAo (*p* = 0.764, 0.516 and 0.053, respectively) but lower axial and higher circumferential WSS components (*p* < 0.001 for both, at all aortic levels). Among BAV patients, RN-BAV presented higher IRF at all levels (*p* = 0.024 proximal, 0.046 mid and 0.002 distal AAo) and higher circumferential WSS at mid and distal AAo (*p* = 0.038 and 0.046, respectively) than RL-BAV. However, axial WSS was higher in RL-BAV compared to RN-BAV at proximal and mid AAo (*p* = 0.046, 0.019, respectively). Displacement and axial WSS were independently associated with the root-morphotype, and circumferential WSS and SFRR with the ascending-morphotype.

**Conclusions:**

Different BAV-phenotypes present different flow patterns with an anterior distribution in RL-BAV, whereas, RN-BAV patients present a predominant posterior outflow jet at the sinotubular junction that shifts to anterior or right anterior in mid and distal AAo. Thus, RL-BAV patients present a higher axial WSS at the aortic root while RN-BAV present a higher circumferential WSS in mid and distal AAo. These results may explain different AAo dilatation morphotypes in the BAV population.

**Electronic supplementary material:**

The online version of this article (10.1186/s12968-018-0451-1) contains supplementary material, which is available to authorized users.

## Background

Bicuspid aortic valve (BAV) is the most common congenital valvular abnormality, occurring in 1–2% of the general population [1]. Between 60 and 80% BAV patients develop aortic dilatation that is associated with an increased risk of aortic dissection and rupture [2, 3]. Aortic diameter alone has proved to be largely ineffective to predict these complications [4–6].

The most common BAV fusion phenotype involves the right and left cusps (RL-BAV) and is associated with dilatation of the tubular ascending aorta (AAo) and aortic root primarily along the convexity of the aorta. While the fusion of the right and non-coronary cusps (RN-BAV) induces arch dilatation with involvement of the tubular ascending aorta, with relative sparing of the root [3]. However, not all patients with the same BAV phenotype have the same pattern of aortopathy and, furthermore, 26–35% of BAV present a non-dilated aorta [2]. Therefore, other factors beyond valve phenotype may be related to aortic dilatation. Although controversy exists regarding the influence of hemodynamic [7, 8] and genetic factors in aortic dilatation [9], different studies have provided significant evidence that altered outflow pattern is related to aortic morphology [7, 10].

In recent years, time-resolved three-dimensional phase-contrast cardiovascular magnetic resonance (CMR 4D-flow) has emerged as a potential tool to provide comprehensive information on aortic hemodynamics with 3D visualization of blood-flow patterns [11, 12]. Using 4D-flow, several studies have analyzed flow and wall shear stress (WSS) on specific analysis planes in the ascending aorta [7, 11, 13, 14], and their relation to aortic dilatation [7]. Although some studies have analyzed WSS components [11, 13], their eventual association to different dilatation morphotype has not been investigated [11]. Only one study analyzed a BAV population differentiating between dilatation morphotype, but the computed WSS was not referenced to local direction [7]. Additionally, it has recently been shown that regions with increased WSS correspond to extracellular matrix dysregulation and elastic fiber degeneration in the ascending aorta and contribute to the development of aortopathy [8, 15, 16]. Thus, a more detailed 3D representation of WSS components [12, 17] may help to explain the different aortic dilatation morphotypes. However, there is not yet sufficient evidence to include these variables for clinical management [7, 11].

The aim of our study was to assess the relation between aortic flow patterns and axial and circumferential WSS by 4D flow CMR through the entire ascending aorta in a large BAV population, and to establish their association with aortic dilatation and morphotype.

## Methods

### Study population

Patients with RL- or RN-BAV phenotype, aortic root and AAo diameters ≤45 mm and no severe valvular disease (aortic regurgitation ≤ Grade III; aortic velocity < 3 m/s) by echo were consecutively and prospectively recruited. Inclusion criteria were: age > 18 years, no cardiovascular disease, sinus rhythm, no hypertension, no connective tissue disorders, no aortic coarctation or other congenital heart diseases and no contraindication for CMR. Also, 20 healthy subjects matched with the BAV population in age and aortic diameters were studied. The study was approved by the local ethics committee and informed consent was obtained from all participants.

### Cardiovascular magnetic resonance protocol

CMR studies were performed on a 1.5 T scanner (Signa, General Electric Healthcare, Waukesha, Wisconsin, USA). The protocol included 2D balanced steady-state free-precession (bSSFP) cine imaging which was used to assess BAV phenotype and aortic diameters (using the double-oblique multiplanar reconstruction), and a 4D-flow acquisition with retrospective electrocardiogram (ECG)-gating during free-breathing. Endovenous contrast was not given.

For 4D-flow CMR, phase-contrast (PC) VIPR sequence [18], a radially undersampled acquisition with 5-point balanced velocity encoding was used [19]. The acquisition volume was set to include the entire thoracic aorta. Acquisitions were made with an eight-channel cardiac coil (HD Cardiac, GE Healthcare) using the following parameters: velocity encoding (VENC) 200 cm/s, field of view (FOV) 400x400x400 mm, scan matrix 160 × 160 × 160 (voxel size of 2.5 × 2.5 × 2.5 mm^3^), flip angle 8°, repetition time 4.2–6.4 ms and echo time 1.9–3.7 ms. This data set was reconstructed the nominal temporal resolution of each patient and was (5xTR) 21 ms–32 ms. Reconstructions were performed offline with corrections for background phase from concomitant gradients and eddy currents, and trajectory errors of the 3D radial acquired k-space [19, 20].

### Data analysis: 4D flow data processing

**Fig. 1:**
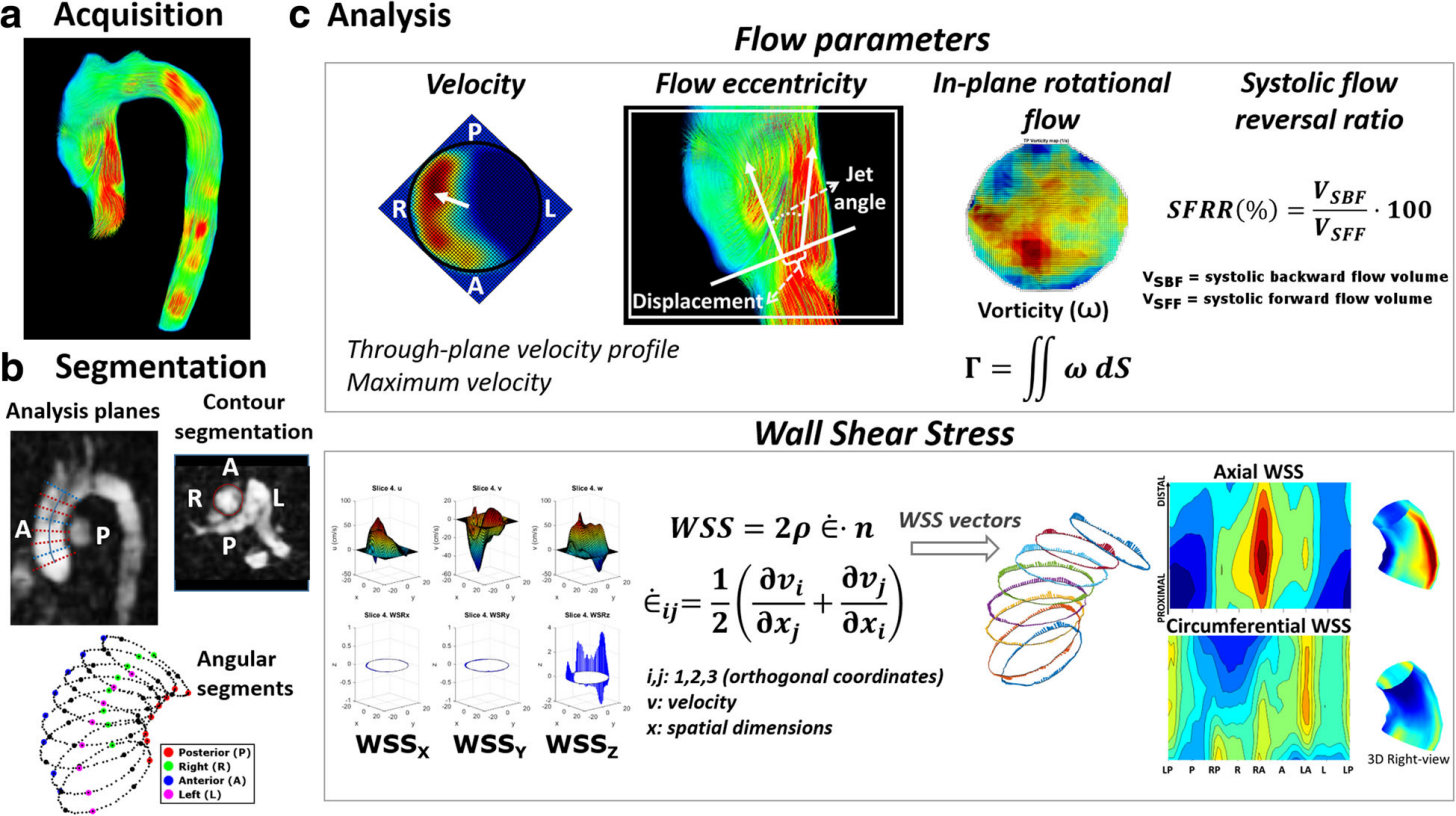
Analysis planes and parameters calculated from 4D-flow CMR (**a**). Eight double-oblique analysis planes were equally distributed in the ascending aorta between the sinotubular junction and the origin of the brachiocephalic trunk (**b**). Velocity profiles, peak velocity, flow eccentricity and in-plane rotational flow were obtained at proximal, mid and distal ascending aorta. Systolic flow reversal ratio was measured at mid and distal ascending aorta. All the analyses planes were used to calculate WSS maps (**c**)

Eight double-oblique analysis planes were equally distributed in the AAo between the sinotubular junction and the origin of the brachiocephalic trunk (see red and blue lines on the left side of Fig. 1b). The vessel lumen was manually segmented in every analysis plane for all systolic phases using an angiogram derived from the 4D-flow data using complex difference processing [21]. Mass Research Software (Leiden University Medical Center, Leiden, the Netherlands) was used for the location of the analysis planes and lumen segmentation. Lumen contour points and 3D velocity data for each plane were exported for calculations to be made using custom Matlab software (MathWorks Inc., Natick, Massachusetts, USA).

### Flow parameters

Peak velocity, flow jet angle, normalized flow displacement, and in-plane rotational flow were calculated at 3 different levels at the proximal, mid and distal AAo (blue lines on Fig. 1b). Flow parameters were averaged using 1 time-frame before and 2 frames after peak systole to mitigate noise.

Flow jet angle and normalized flow displacement were obtained as described by Sigovan et al. [22]. In-plane rotational flow was quantified as the through-plane component (Γ_T_) of circulation (Γ), which is a parameter used in fluid dynamics to quantify rotation of flow within a plane. To this aim, vorticity (ω) was computed in each double-oblique analysis plane and circulation (Γ) was obtained as the integral of vorticity with respect to cross-sectional area, Γ =  ∬ *ω dS* [23].

**Fig. 2:**
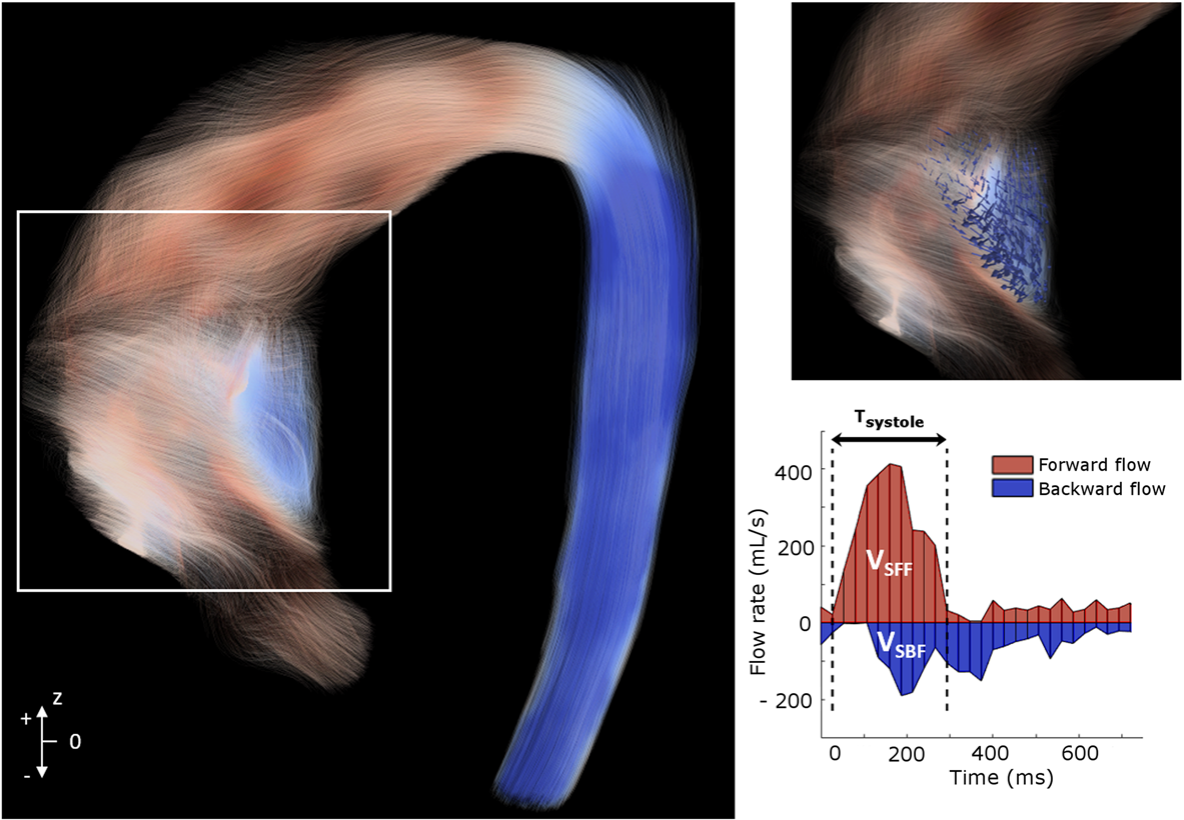
Systolic backward flow in a BAV patient. Red streamlines indicate forward flow in the ascending aorta, while blue streamlines indicate systolic backward flow. SFRR: systolic flow reversal ratio, V_SFF_: total systolic forward flow, V_SBF_: total systolic backward flow

Flow volumes were calculated as the time-integral over systolic phases of forward and backward through-plane flow rate curves, and used for the calculation of systolic flow reversal ratio (SFRR) [24] at mid and distal AAo (Figs. 1 and 2).

$$ SFRR\left(\%\right)=\frac{\int_0^{T_s}{v}_{SBF}(t) dt}{\int_0^{T_s}{v}_{SFF}(t) dt}\cdot 100 $$

Where *v*_*SFF*_ and *v*_*SBF*_ represent the forward and backward flow rates, respectively, and T_s_ is systolic time interval.

### Wall shear stress

Peak-systolic WSS vectors (averaged from 1-time frame before, and 2-time frames after peak) were calculated at 64 points equally distributed along the aortic lumen for the 8 cross sectional analysis planes by fitting the 3D velocity data with B-spline surfaces and computing velocity derivatives on the segmented vessel lumen [25]. WSS vectors were decomposed in their through-plane (axial) and in-plane (circumferential) components.

Contour-averaged magnitude (WSS_mag,avg_) and WSS components (WSS_ax,avg_ and WSS_circ,avg_) were calculated at proximal, mid and distal AAo.

Averaged WSS maps were obtained for each BAV phenotype and dilatation morphotype (Figs. 1c, 6 and 8). To this aim, the 64 points of the lumen contour per cross-sectional plane were aligned for all patients using the inner aortic curvature as a reference. Averaged WSS maps were calculated by computing point-to-point WSS means for all the 8 sections analyzed. Finally, statistical significance maps of axial and circumferential WSS were calculated for the mean WSS value for 8 standardized angular segments [14] of the aortic wall. Averaged WSS maps and statistical significance maps were visualized using bilinear interpolation.

### Dilatation morphotypes

In order to determine the presence of aortic root or ascending dilatation, aortic diameters were adjusted with a logarithm transformation to set the z-score for both sinuses (zsinus) and ascending aorta (zAscAo) accounting for sex, age and body surface area (BSA) as described by Campens et al. [26]. Using a z-score cutoff for aortic dilatation of 2 standard error of estimate, patients were categorized according to the tract predominantly or exclusively involved in dilatation according to Della Corte’s classification [2]. Thus, patients were classified as non-dilated (zsinus≤2 and zAscAo≤2), root-morphotype (zsinus> 2 and zsinus>zAscAo) or ascending-morphotype (zAscAo> 2 and zAscAo>zsinus).

### Statistical analysis

Continuous demographic variables were expressed as mean ± standard deviation. The Kolmogorov-Smirnov test was used to evaluate the normality distribution of variables. Differences between groups for continuous parameters were assessed by Student’s t-test if they presented a normal distribution or ANOVA with Bonferroni correction for multiple comparisons, and Mann-Whitney U test if they did not present a normal distribution. For categorical variables, general characteristics of the sample were assessed by percentages (chi-square test). Logarithmic transformation (ln) was performed for variables with both positive and negative values (such as circulation and WSS) to preserve the distinction between negative, zero and positive values as described by Whittaker et al. [27].

Multivariable logistic regression analyses with a forward selection procedure were used to evaluate specific relations between demographic and flow variables and aortic root or ascending dilatation. Variables were entered into the model if *P* < 0.25 in univariate analyses. The aortic root morphotype was compared to the rest of groups (non-dilated and ascending), whereas, the ascending group was compared to non-dilated and root morphotype as described elsewhere [28]. To avoid multicollinearity, variables were excluded from the multivariable logistic regression if the tolerance test was < 0.1 or the variation inflation factor > 5. This is the case of the same flow variables computed at different locations. The variables entered the multivariable model were chosen as those demonstrating better predictive value, i.e. those having higher AUC in the Receiver operating characteristic (ROC) curve, as compared to the same variable computed at another location. ROC curves were performed to assess the relationship between variables obtained in the multivariable analysis and aortic morphotypes.

A two-tailed *P* value < 0.05 was considered statistically significant. SPSS (version 19.0, International Business Machines, Armonk, New York, USA) was used for the analysis.

## Results

One hundred and one BAV patients (78 RL- and 23 RN-phenotype) and 20 healthy subjects completed the study protocol. Demographic characteristics and aortic diameters among groups are shown in Table 1. Demographics did not significantly differ among the three groups (controls, RN-BAV, RL-BAV). Although aortic diameters were larger in BAV compared to controls, no statistically-significant differences were observed. However, z-scores were higher in the RL-BAV phenotype at the sinuses of Valsalva and at the AAo in the RN-BAV.

**Table 1:** Demographics and aortic dimensions in controls and bicuspid aortic valve patients

	Healthy Controls	RL-BAV	RN-BAV	*p*-value
*N*	20	78	23	
Age (years)	50.04 ± 16.39	48.43 ± 13.15	46.97 ± 16.00	0.659
Men (%)	73.30	62.80	65.20	0.736
Weight (kg)	78.57 ± 9.24	76.17 ± 10.38	76.17 ± 10.38	0.270
Height (cm)	169.42 ± 7.73	170.00 ± 10.66	172.00 ± 9.91	0.433
Body Surface Area (m^2^)	1.89 ± 0.13	1.83 ± 0.21	1.88 ± 0.16	0.278
Degree of Aortic Regurgitation (%)				0.240
0	–	26.8	35.3	
1	–	21.1	5.9	
2	–	49.3	47.0	
3	–	2.8	11.8	
Degree of Aortic Stenosis (%)				0.695
Absent	–	92.9	90	
Mild	–	5.7	10	
Moderate	–	1.4	0	
Maximum aortic velocity (m/s)	97.34 ± 19.0	111.15 ± 24.35	119.32 ± 21.22	0.067
Mean pressure gradient (mm Hg)	3.24 ± 1.21	4.9 ± 1.13	5.69 ± 1.71	0.074
Systolic Arterial Pressure (mm Hg)	136.26 ± 19.47	134.18 ± 17.32	142 ± 17.76	0.064
Diastolic Arterial Pressure (mm Hg)	76.73 ± 8.85	75.72 ± 9.06	79.69 ± 9.11	0.133
Sinus of Valsalva Diameter (mm)	32.77 ± 3.44	36.68 ± 5.10	34.78 ± 2.69	0.796
Ascending Aorta Diameter (mm)	35.58 ± 2.96	39.86 ± 7 .51	40.30 ± 6.10	0.070
z-score sinus of Valsalva diameter	0.01 ± 0.84	1.41 ± 1.29	0.82 ± 1.11	0.001
z-score ascending aorta diameter	0.02 ± 0.71	2.87 ± 1.71	3.03 ± 1.4	< 0.001

*P*-values reported are the result of ANOVA comparison between 3 groups: controls, RL-BAV and RN-BAV in continuous variables and Chi-square test for categorical variables

In the RL-BAV group (*n* = 78), 20 patients (25.6%) presented a non-dilated, 10 (12.8%) a root- and 48 (61.5%) an ascending-morphotype aorta. In the RN-BAV group (*n* = 23), 4 patients (17.4%) presented a non-dilated, 1 (4.3%) a root- and 18 (78.3%) an ascending-morphotype. Only three of the patients with root-morphotype did not present ascending aorta dilatation (zAscAo< 2). These root-only dilated patients were all men (mean age 40 years), RL-BAV and presented different degrees of aortic regurgitation and no aortic stenosis.

### Peak velocity and flow eccentricity

**Fig. 3:**
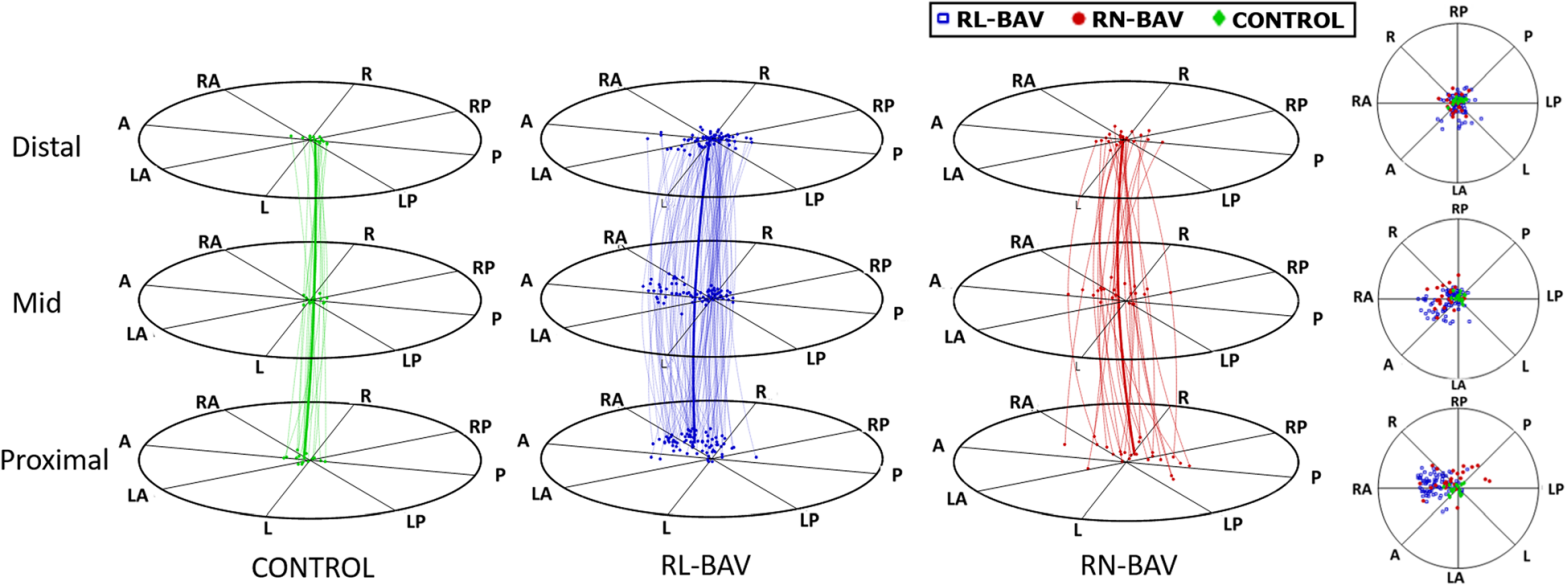
Peak-systolic center of velocities. Center of velocities at proximal, mid and distal ascending aorta (AAo) for controls and BAV. Lines show the maximum velocity path along the AAo for each patient. Wider arrow represents the mean path for each group. A: anterior, L: left, P: posterior, R: right, RL-BAV: right-left bicuspid aortic valve, RN-BAV: right-non coronary bicuspid aortic valve

Through-plane and magnitude velocity, jet angle and normalized displacement at proximal, mid and distal AAo are shown in Table 2. Compared to healthy control subjects, BAV patients presented higher through-plane velocity values at proximal AAo, and higher velocity magnitude, jet angle and flow displacement at the proximal and mid AAo but not in the distal part (Fig. 3). Also, RN- compared to RL-BAV presented significant differences in peak velocity and eccentricity at proximal AAo.

**Table 2:** Flow dynamics in controls and BAV patients depending on BAV phenotype and dilatation morphotype

		Total population			BAV phenotype			Ascending aorta morphotype (only BAV)			
Plane	Measurements	CONTROLS (*n* = 20)	BAV (*n* = 101)	*p*	RL-BAV (*n* = 78)	RN-BAV (*n* = 23)	*p*	Non-dilated (*n* = 24)	Root (*n* = 11)	Ascending (*n* = 66)	*p*
Prox	Peak velocity magnitude (cm/s)	111.7 ± 21.9	133.9 ± 28.3	0.004	128.5 ± 27.7	152.4 ± 22.2	0.001	122.6 ± 26.8	134.0 ± 23.9	138.0 ± 28.8	0.073
	Peak TP velocity (cm/s)	97.3 ± 19	113.0 ± 23.8	0.020	111.2 ± 24.4	119.3 ± 21.2	0.112	106.8 ± 26.1	113.4 ± 22.0	115.2 ± 23.2	0.343
	Jet angle (°)	24.3 ± 6.2	25.7 ± 12.1	0.666	23.3 ± 11.3	33.8 ± 11.8	< 0.0001	21.1 ± 14.0	24.2 ± 13.8	27.7 ± 10.8	0.042
	Normalized displacement	0.04 ± 0.02	0.15 ± 0.07	< 0.001	0.15 ± 0.06	0.12 ± 0.06	0.045	0.10 ± 0.06	0.12 ± 0.05	0.16 ± 0.05	< 0.0001
	IRF (cm^2^/s)	−22.9 ± 65.9	139.1 ± 143.5	< 0.001	123.0 ± 128.4	193.4 ± 178.3	0.024	54.2 ± 124.3	162.3 ± 162.4	166.1 ± 136.5	0.001
	WSS_mag,avg_ (N/m^2^)	0.52 ± 0.09	0.56 ± 0.16	0.764	0.55 ± 0.15	0.57 ± 0.17	0.789	0.53 ± 0.14	0.55 ± 0.18	0.56 ± 0.15	0.608
	WSS_ax,avg_ (N/m^2^)	0.41 ± 0.11	0.18 ± 0.12	< 0.001	0.19 ± 0.11	0.13 ± 0.09	0.046	0.26 ± 0.14	0.21 ± 0.11	0.14 ± 0.08	0.001
	WSS_circ,avg_ (N/m^2^)	−0.03 ± 0.11	0.30 ± 0.20	< 0.001	0.29 ± 0.18	0.31 ± 0.35	0.590	0.18 ± 0.22	0.32 ± 0.20	0.33 ± 0.18	0.007
Mid	Peak velocity magnitude (cm/s)	93.1 ± 24.3	118.8 ± 31.1	0.002	116.8 ± 32.3	125.8 ± 26.0	0.232	110.4 ± 22.7	114.5 ± 30.1	122.6 ± 33.5	0.282
	Peak TP velocity (cm/s)	89.7 ± 24.3	100.7 ± 27.7	0.060	101.0 ± 30.3	99.5 ± 16.5	0.833	99.0 ± 22.7	95.8 ± 22.8	102.0 ± 30.1	0.877
	Jet angle (°)	10.2 ± 5.9	28.7 ± 11.4	< 0.001	28.3 ± 12.0	30.1 ± 8.8	0.384	23.0 ± 10.6	25.4 ± 10.3	31.4 ± 11.0	0.001
	Normalized displacement	0.03 ± 0.01	0.09 ± 0.06	< 0.001	0.09 ± 0.06	0.09 ± 0.04	0.639	0.07 ± 0.06	0.06 ± 0.04	0.11 ± 0.06	0.001
	IRF (cm^2^/s)	35.1 ± 50.7	199.6 ± 190.3	< 0.001	186.2 ± 177.4	245.0 ± 227.4	0.046	101.1 ± 94.4	190.8 ± 200.2	236.9 ± 203.5	0.003
	WSS_mag,avg_ (N/m^2^)	0.54 ± 0.15	0.58 ± 0.21	0.516	0.57 ± 0.21	0.59 ± 0.22	0.907	0.55 ± 0.16	0.56 ± 0.21	0.59 ± 0.23	0.888
	WSS_ax,avg_ (N/m^2^)	0.46 ± 0.14	0.29 ± 0.14	< 0.001	0.30 ± 0.13	0.23 ± 0.11	0.019	0.37 ± 0.17	0.30 ± 0.12	0.25 ± 0.10	0.010
	WSS_circ,avg_ (N/m^2^)	0.06 ± 0.07	0.29 ± 0.22	< 0.001	0.27 ± 0.20	0.34 ± 0.26	0.038	0.17 ± 0.17	0.28 ± 0.24	0.33 ± 0.21	0.004
	SFRR (%)	3.99 ± 3.74	20.9 ± 13.5	< 0.001	21.4 ± 14.6	19.2 ± 8.7	0.777	12.3 ± 12.7	15.0 ± 11.7	25.0 ± 12.3	< 0.0001
Dist	Peak velocity magnitude (cm/s)	96.3 ± 21.6	106.1 ± 30.0	0.195	105.0 ± 30.2	109.8 ± 29.4	0.439	105.7 ± 20.4	107.5 ± 30.7	106.0 ± 33.0	0.991
	Peak TP velocity (cm/s)	87.4 ± 18.1	85.6 ± 23.6	0.886	86.7 ± 23.3	81.8 ± 24.7	0.388	91.1 ± 19.0	88.3 ± 22.4	83.1 ± 25.1	0.374
	Jet angle (°)	23.0 ± 9.0	24.0 ± 10.6	0.690	25.1 ± 10.8	20.4 ± 9.5	0.138	19.5 ± 6.6	24.1 ± 9.1	25.6 ± 11.6	0.051
	Normalized displacement	0.03 ± 0.01	0.06 ± 0.04	0.027	0.06 ± 0.04	0.05 ± 0.03	0.749	0.05 ± 0.03	0.06 ± 0.05	0.06 ± 0.04	0.813
	IRF (cm^2^/s)	36.6 ± 35.3	120.9 ± 140.0	0.013	96.2 ± 117.8	204.7 ± 176.1	0.002	60.7 ± 70.8	106.1 ± 178.3	145.2 ± 146.7	0.005
	WSS_mag,avg_ (N/m^2^)	0.58 ± 0.13	0.50 ± 0.18	0.053	0.48 ± 0.16	0.53 ± 0.20	0.333	0.48 ± 0.15	0.53 ± 0.18	0.49 ± 0.18	0.767
	WSS_ax,avg_ (N/m^2^)	0.36 ± 0.13	0.24 ± 0.12	0.001	0.25 ± 0.12	0.22 ± 0.10	0.356	0.28 ± 0.16	0.27 ± 0.11	0.22 ± 0.09	0.082
	WSS_circ,avg_ (N/m^2^)	0.07 ± 0.05	0.22 ± 0.19	< 0.001	0.19 ± 0.17	0.29 ± 0.22	0.046	0.13 ± 0.13	0.19 ± 0.24	0.25 ± 0.19	0.020
	SFRR (%)	2.63 ± 3.47	10.9 ± 9.0	< 0.001	11.5 ± 9.6	9.1 ± 5.8	0.469	9.3 ± 11.6	8.3 ± 5.5	11.9 ± 8.3	0.026

Values are mean ± SD

*TP* through-plane, *BAV* bicuspid aortic valve, *RL* right-left, *RN* right-non coronary, *IRF* in-plane rotational flow, *WSS* wall shear stress, *WSSmag, avg.* contour-averaged WSS magnitude, *WSSax, avg.* contour-averaged axial WSS, *WSScirc, avg.* contour-averaged circumferential WSS, *SFRR* systolic flow reversal ratio

When the aortic morphotype was considered in BAV, the ascending and root-morphotypes (dilated morphotype) compared to the non-dilated presented higher jet angles and displacement at the proximal and mid AAo (*P* < 0.05). However, the ascending versus the root-morphotypes did not present differences (Table 2).

**Fig. 4:**
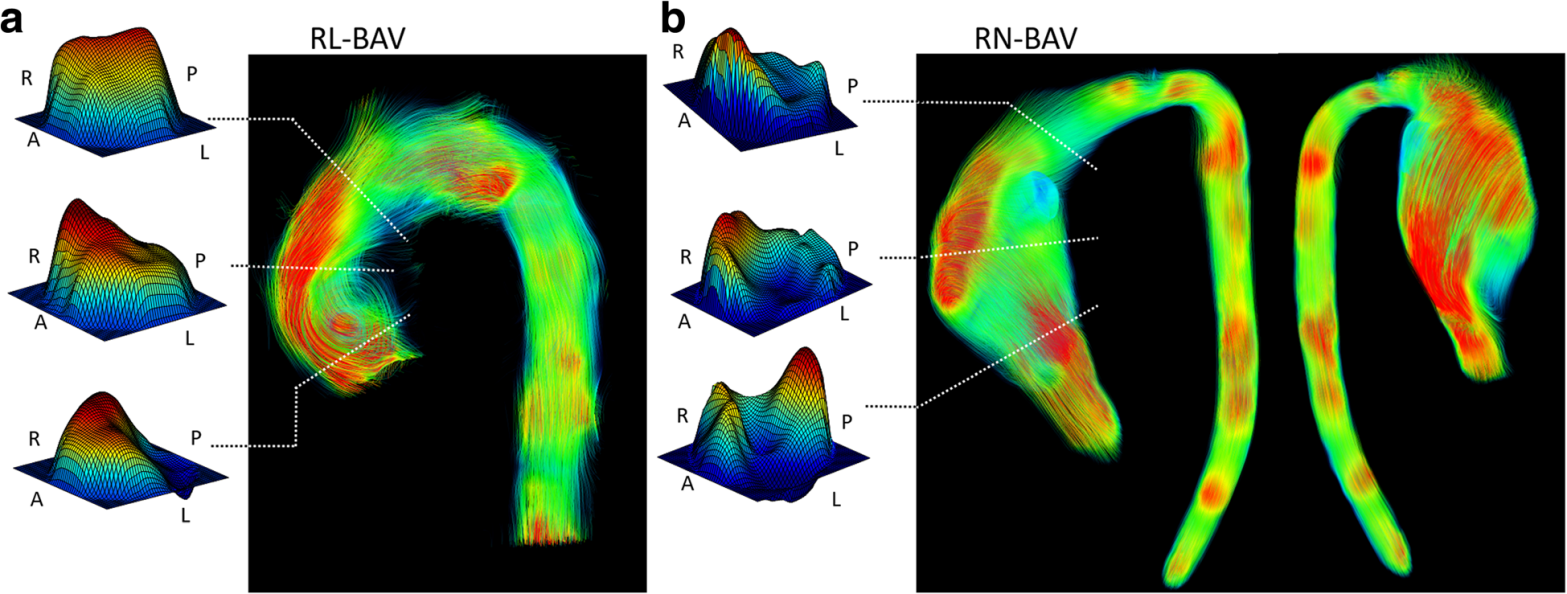
Through-plane velocity profiles and streamlines in BAV. **a** Asymmetric outflow jet to the anterior wall in a RL-BAV. **b** RN-BAV with a posterior outflow jet shifting to the anterior wall at mid and distal ascending aorta. Abbreviations as in Fig. 3

When the location of the center of velocities and flow direction were analyzed in BAV, RL-BAV presented a consistent pattern (Fig. 3), showing a right (28% cases) to right-anterior position (55% cases) in the proximal aorta, with a similar profile in the mid segment and an axisymmetric profile at the distal AAo (Fig. 4a and additional movie file [Additional file 1]). However, RN-BAV presented a higher variability in their flow pattern (Fig. 3) with a predominant posterior to right-posterior outflow jet (78% cases) in the proximal aorta that shifted to the anterior segments (55% right-anterior and 20% anterior) in the mid and distal AAo (Fig. 4b and additional movie file [Additional file 2]). Control patients presented non-eccentric and predominantly laminar flow (see additional movie file [Additional file 3]).

### In-plane rotational flow and systolic flow reversal ratio

In-plane rotational flow (IRF) was significantly higher in RN- compared to RL-phenotype at all aortic levels. Although a trend for higher SFRR was observed in RL-BAV compared to RN-BAV, these differences did not reach statistical significance (Table 2).

**Fig. 5:**
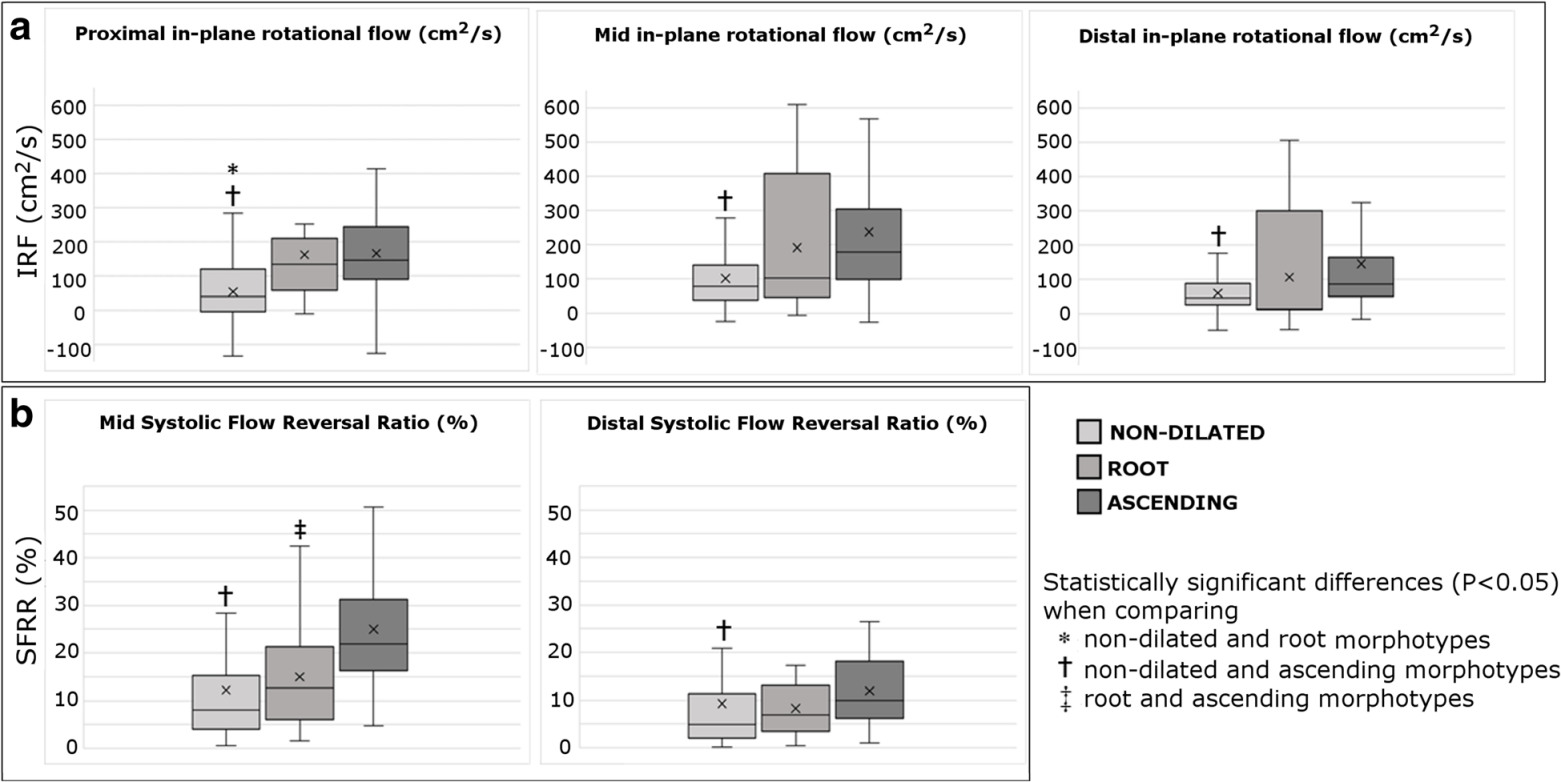
In-plane rotational flow (IRF) and systolic backward flow (SFRR). **a** Mean IRF and **b** SFRR in controls and BAV, based on the dilatation morphotype

Dilated BAV presented higher values of IRF and SFRR compared to healthy controls or non-dilated morphotype (Table 2, Fig. 5). IRF was mostly right-handed (98%) in all aortic morphotypes with no statistically-significant differences. However, patients with the ascending-morphotype presented higher IRF and SFRR than the root-morphotype at all aortic levels (Table 2) (Fig. 5).

### WSS and regional WSS maps

Compared to controls, BAV presented similar magnitude (WSS_mag,avg_) (*P* > 0.05) but lower axial (WSS_ax,avg_) and higher circumferential WSS (WSS_circ,avg_) at all levels (*P* < 0.001) (Table 2).

**Fig. 6:**
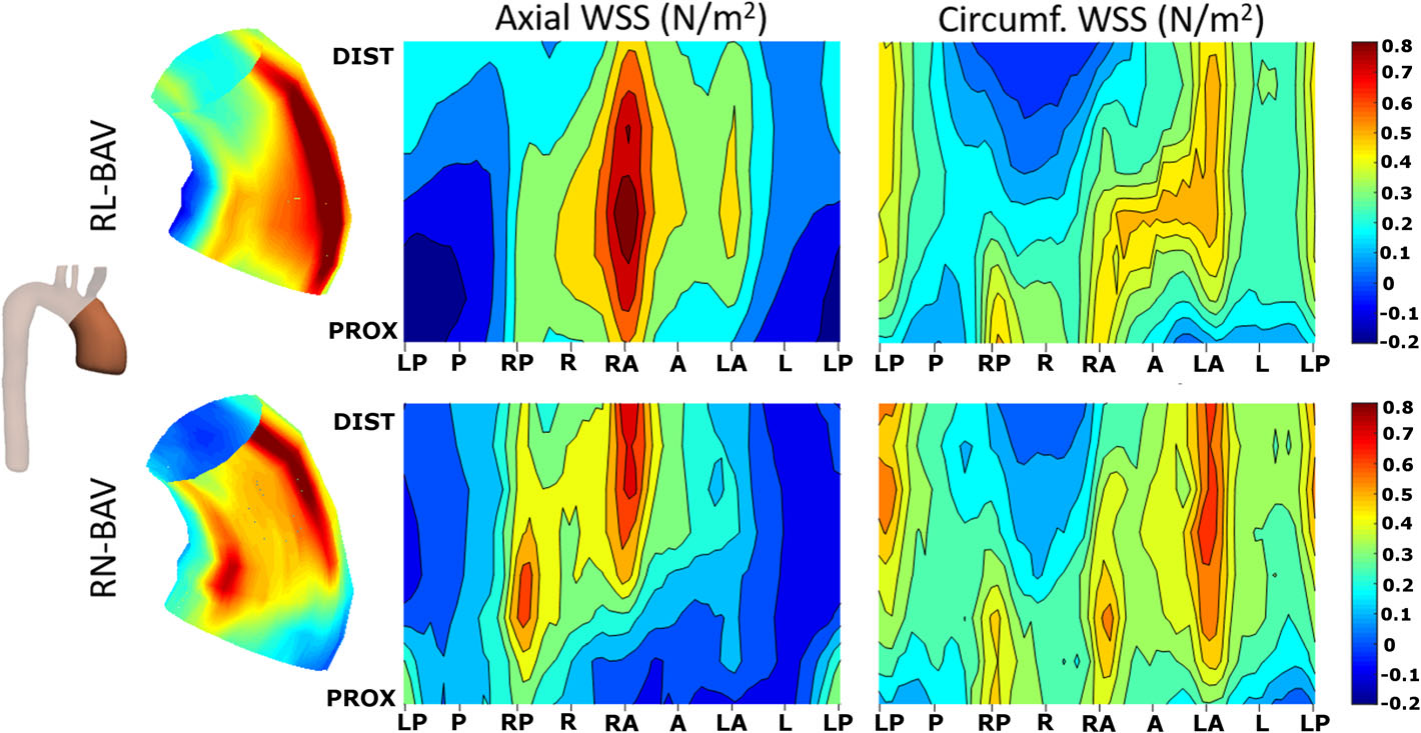
Peak-systolic axial and circumferential WSS maps in RL- and RN-BAV. RL-BAV show maximum axial WSS in the right to right-anterior wall and lower values of circumferential WSS at all levels. In RN-, maximum axial WSS extends from the right-posterior wall proximally to right-anterior wall at mid and distal ascending aorta, with a higher distal circumferential WSS. Prox: proximal, Dist: distal, WSS: wall shear stress, other abbreviations as in Fig. 3

**Fig. 7:**
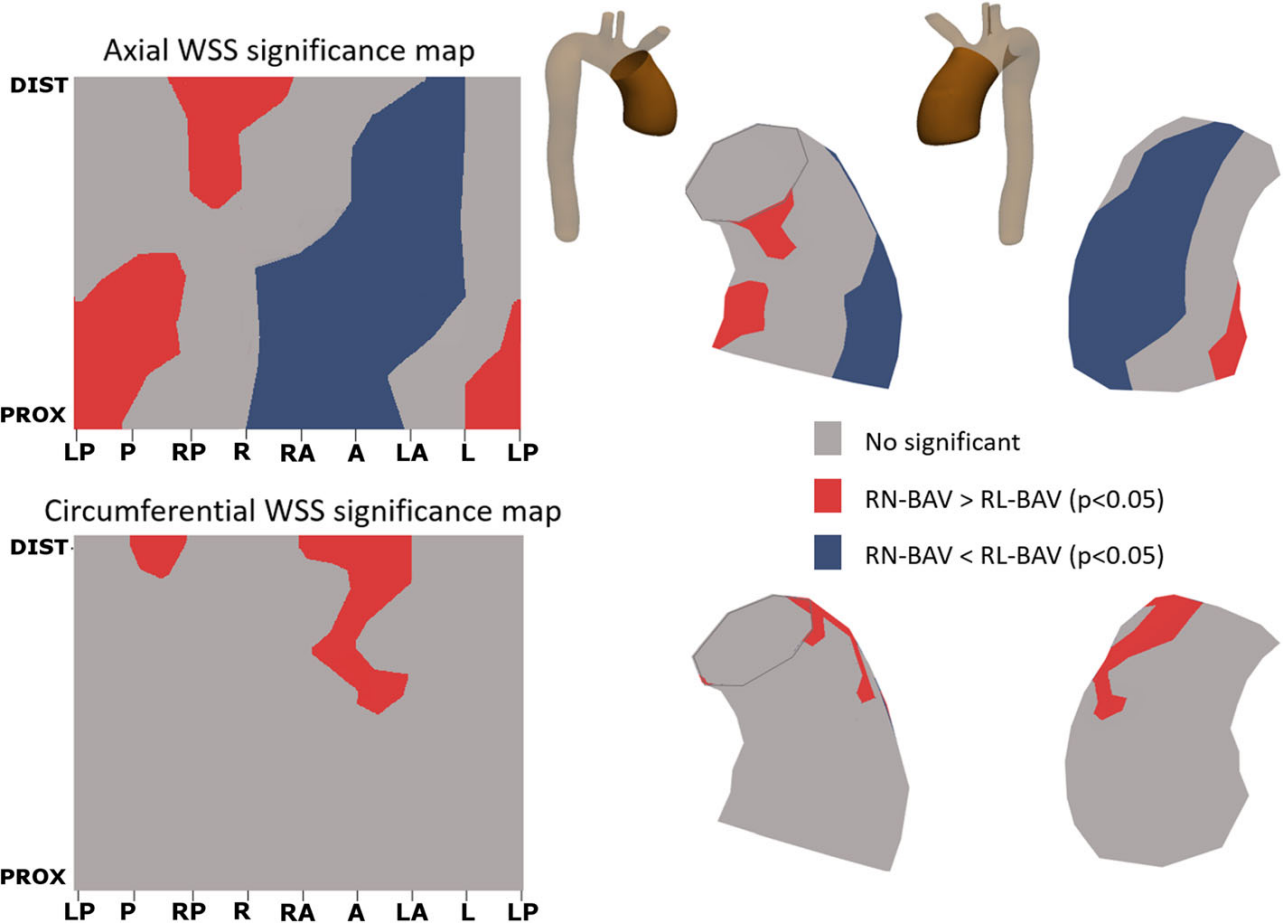
Statistical significance maps of axial and circumferential WSS comparing BAV phenotypes. Axial WSS regions correlate well with jet direction in BAV phenotypes. Circumferential WSS is significantly higher in the distal ascending aorta for RN-BAV patients. Abbreviations as in Fig. 3

According to the valve phenotype, RL- compared to RN-BAV presented higher WSS_ax,avg_ in proximal and mid AAo (*P* < 0.05), whereas WSS_circ,avg_ was higher in RN-phenotype in mid and distal AAo (Table 2). These differences were also observed in regional WSS maps (Figs. 6 and 7).

**Fig. 8:**
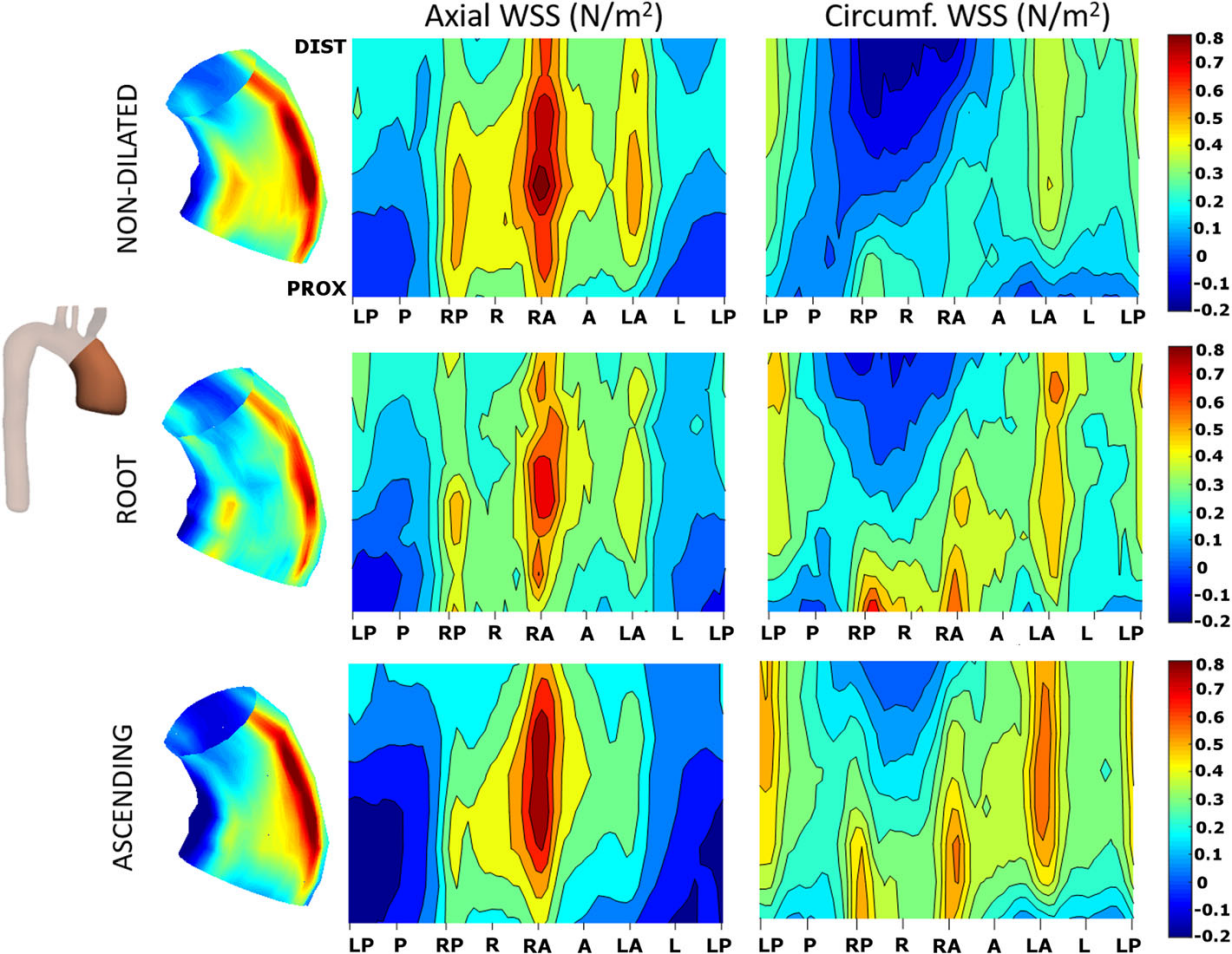
Peak-systolic axial and circumferential WSS maps according to BAV aortic morphotype. Root-morphotype presents higher proximal axial WSS compared to ascending-, however, circumferential WSS is higher in ascending-morphotype at all levels. Abbreviations as in Fig. 3

**Fig. 9:**
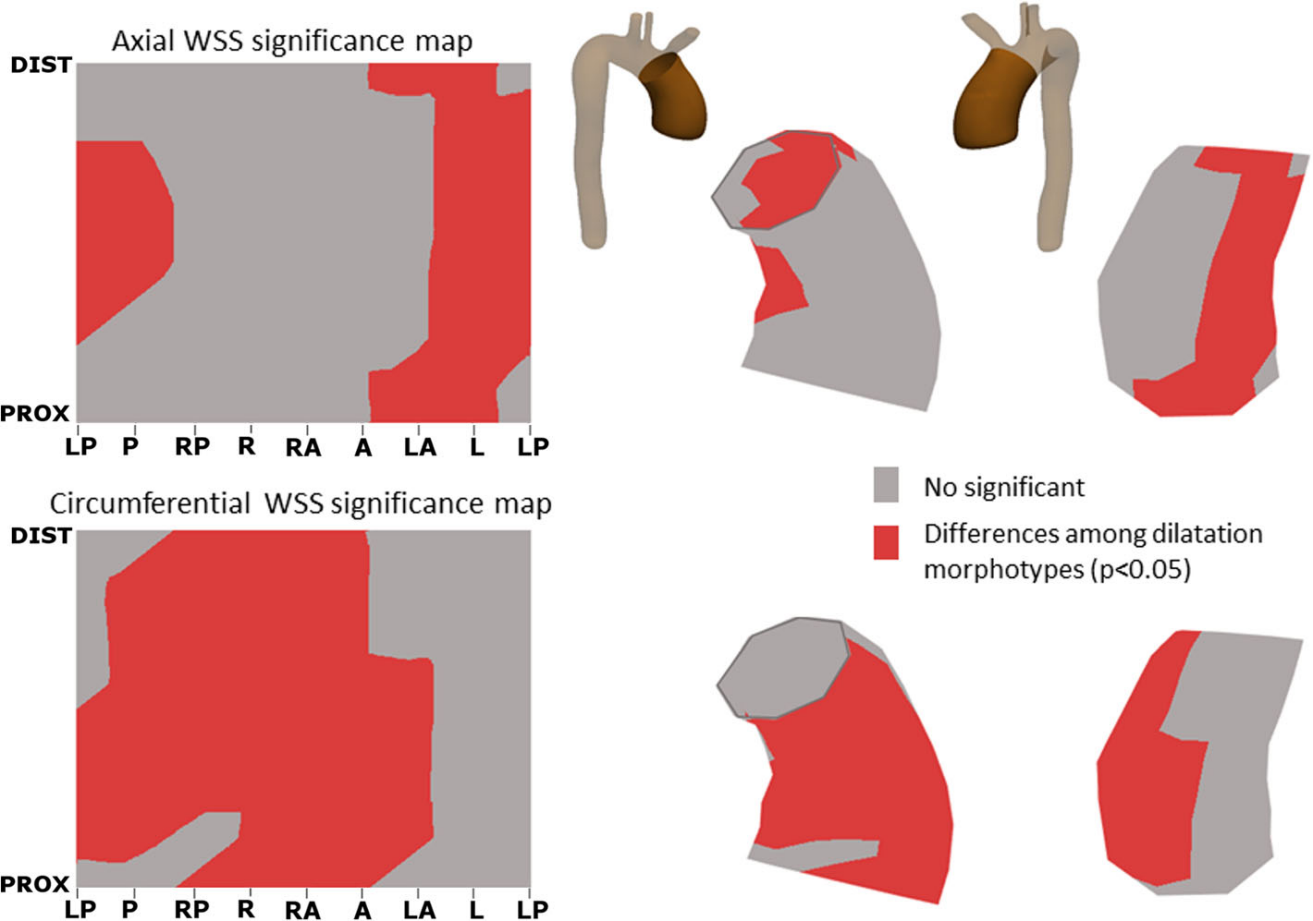
Statistical significance maps of axial and circumferential WSS comparing BAV morphotypes. Statistical differences in regional WSS are more pronounced in circumferential than axial WSS, and are associated with regions of systolic flow reversal for axial WSS

Based on the aortic morphotype, non-dilated BAV presented similar WSS_mag avg_ and WSS_ax,avg_ but higher WSS_circ,avg_ compared to controls at all levels (*P* < 0.05). In BAV patients, the WSS_mag,avg_ was similar among the different aortic morphotypes. However, WSS_ax,avg_ was significantly higher in the root-morphotype and WSS_circ,avg_ was higher in the ascending-morphotype at all levels (Table 2). Regional differences (*p* < 0.05) between morphotypes were more pronounced for circumferential than axial WSS, and were associated with regions of systolic flow reversal for axial WSS (Figs. 8 and 9).

The outflow jet direction matched the location of maximum systolic axial WSS in the aortic wall (Figs. 3 and 6). Thus, in RN-BAV, maximum systolic axial WSS extends from posterior to right-posterior proximally towards anterior to right-anterior in mid and distal AAo. However, in RL-BAV, maximum systolic axial WSS extends from right to right-anterior at all aortic levels (Figs. 6 and 7).

### Correlates of root or ascending-morphotypes

Significant bivariable (unadjusted) and multivariable adjusted correlates of aortic dilatation (> 2 z-score) [26] in BAV are listed in Table 3 (variables selected from the 4D-flow-derived variables listed in Table 2 and including sex, age and body surface area). Displacement, IRF and WSS were transformed to their natural logarithms.

**Table 3:** Unadjusted and adjusted relationship of demographic and flow variables to root (top) and ascending (bottom) morphotypes

Root morphotype	Univariate adjusted correlates of root morphotype		Multivariable adjusted correlates of root morphotype	
	OR	*p*-value	OR	*p*-value
Age	0.99 (0.96–1.02)	0.530		
BSA	0.17 (0.02–1.45)	0.104	0.08	0.291
BAV phenotype (RN vs RL)	1.41 (1.17–2.28)	0.005	1.35	0.998
Sex (male)	1.59 (1.32–1.95)	0.050	1.25	0.011
Proximal peak velocity magnitude (cm/s)	1.01 (0.98–1.22)	0.719		
Proximal jet angle (°)	1.03 (0.95–1.51)	0.490		
Ln Proximal normalized displacement	1.98 (1.01–2.93)	0.040	1.1	0.009
Ln Proximal IRF (cm^2^/s)	1.22 (1.02–1.45)	0.002	0.79	0.940
Ln Proximal WSS_mag,avg_ (N/m^2^)	1.54 (0.09–4.52)	0.761		
Ln Proximal WSS_ax,avg_ (N/m^2^)	1.621 (0.93–2.95)	0.081	1.13	0.003
Ln Proximal WSS_circ,avg_ (N/m^2^)	2.76 (0.41–4.71)	0.243	0.02	0.673
Ascending morphotype	Univariate adjusted correlates of ascending morphotype		Multivariable adjusted correlates of ascending morphotype	
	OR	*p*-value	OR	*p*-value
Age	0.99 (0.96–1.03)	0.917		
BSA	2.51 (0.30–2.17)	0.398		
BAV phenotype (RN vs RL)	1.99 (1.61–3.48)	0.026	1.73	0.04
Sex (male)	0.82 (0.32–2.10)	0.678		
Mid jet angle (°)	1.09 (1.03–1.14)	0.001	1.025	0.353
Ln Mid normalized displacement	2.85 (1.56–5.23)	0.001	1.510	0.270
Ln Mid IRF (cm^2^/s)	2.98 (1.54–5.78)	0.001	1.195	0.802
Ln Mid WSS_ax,avg_ (N/m^2^)	0.14 (0.04–0.48)	0.221	0.960	0.950
Ln Mid WSS_circ,avg_ (N/m^2^)	1.51 (1.16–3.68)	0.002	1.61	0.011
Mid SFRR (%)	1.12 (1.06–1.19)	0.001	1.11	0.001

**Fig. 10:**
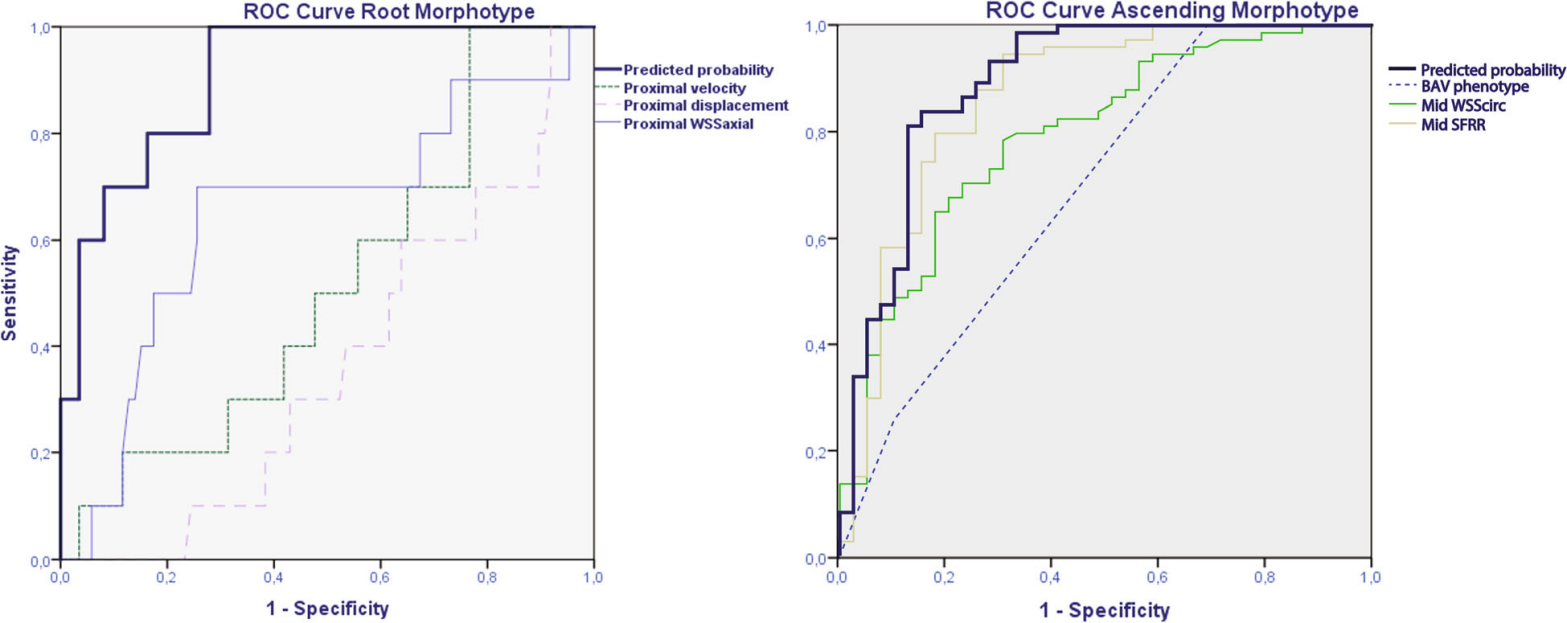
ROC curves showing main factors associated with ascending aorta morphotypes. Composite probability shows the best AUC for prediction in both morphotypes. SFRR: systolic flow reversal ratio, WSScirc: circumferential WSS

On multivariable analysis, only sex (male), natural logarithms of displacement and WSS_ax,avg_ were related to the presence of the root-morphotype with an AUC: 0.91 (*P* < 0.001) (Fig. 10). However, RN-phenotype, SFRR and WSS_circ,avg_ in the mid AAo were related to the presence of the ascending- morphotype with an AUC: 0.89 (*P* < 0.001) (Table 3) (Fig. 10).

## Discussion

In our study, we assessed the relation between aortic flow patterns and regional WSS components (magnitude, axial and circumferential) through the entire ascending aorta in a large BAV population. In order to avoid the effect of changes in flow dynamics and WSS secondary to aortic dilatation [29] or severe valvular disease, only BAV patients with non-severe valvular dysfunction and aortic diameters ≤45 mm were included. Also, the specific role of flow parameters and WSS components in the ascending aorta dilatation and morphotype was assessed by unadjusted and multivariable adjusted analysis.

The main findings of our study were that: 1) RL-BAV patients present a sustained flow towards the anterior and right-anterior aortic walls, whereas, RN-BAV present a predominantly posterior output flow that shifts towards the right and right-anterior walls in the mid and distal AAo inducing an increase in the IRF. This flow distribution reflects into regional WSS patterns. 2) Sex (male), normalized displacement and axial WSS in the proximal AAo are the main factors associated with the root-morphotype, whereas RN-phenotype, SFRR and circumferential WSS are the main factors related to the ascending-morphotype.

To our knowledge, this is the first large study conducted in BAV patients in which different patterns of axial and circumferential regional WSS maps were used to explain variations in the AAo dilatation morphotype. Previous studies have emphasized differences in flow variables and WSS between the RN- and RL-BAV [7, 10, 11, 13, 14] with little or no correlation with the aortic dilatation morphotype [7, 11]. In this regard, Mahadevia et al. [7] did not analyze rotational flow or WSS components when studying BAV aortopathy, while Bissell et al. [11] only showed an increased rotational flow at larger aortic diameters suggesting a potential causative role. Thus, it is of great interest to ascertain the main factors associated with aortic dilatation since aortic diameter and structural changes in the aortic wall are related to clinical events regardless of valve phenotype [6, 8].

### Flow patterns

Owing to the asymmetric valve opening, there is an increase in the jet angle and in the displacement of the center of velocities with respect to the center of the lumen that induces an asymmetric distribution of the WSS pattern as previously described [7, 11, 13, 14]. Similar to those of Mahadevia et al. [7], our results confirm that the jet angle is wider in RN-phenotype, whereas normalized displacement is greater in the RL-phenotype. Also, we demonstrated that these variables are greater at the proximal aorta with a progressive reduction at the distal AAo that suggests that flow tends to be more symmetric in the distal AAo. Our results differ from those of Mahadevia et al. [7] since they reported flow angle and displacement to be the main factors involved in AAo dilatation. However, they determined the absolute value of the displacement, whereas we report this value normalized by aortic diameter as suggested elsewhere [22].

### In-plane rotational flow and SFRR

Similar to previous studies [11, 13, 30, 31], we found that BAV presented greater rotational flow compared to controls, with the RN-phenotype being greater than in RL- at the mid and distal AAo. This finding can be justified by the fact that the flow shifts from posterior towards anterior segments in RN-BAV. This rotational flow is not only significantly greater in RN-BAV but also in those with the ascending-morphotype. An increased rotational flow induces an increase in the circumferential WSS which justifies that both parameters were statistically significant in the ascending-morphotype on univariable analysis.

In our population, most of the BAV patients presented right-handed flow (98%). The lack of left handed helical flow seen in our study could be a sign of left handed helical flow being associated with severe/late disease process [11], and therefore, not seen in the benign aortopathy population (≤ 45 mm) included in our study.

The presence of retrograde flow at systole has been reported in patients with greater aortic diameters [29, 32, 33]. We found that higher values of SFRR are associated with the ascending-morphotype and not with the root-morphotype. An increase in the SFRR may induce an asymmetric increase and directional variations in the WSS contributing to dilatation. It is not clear whether this parameter is the cause or consequence of aortic dilatation; however, we observed that this cranio-caudal flow also exists in BAV with normal aortic diameters. This finding suggests that this flow may act as a causal agent of aortic dilatation and would increase as the aorta dilates, thereby perpetuating this process.

### WSS and aortic dilatation

Our study is consistent with previous publications [7, 13] which suggest that the magnitude of WSS lacks significance since its value is similar in controls and BAV. However, controls present increased axial WSS because of predominant laminar flow, while helical flow in BAV increases circumferential WSS [13]. Thus, the different WSS components (axial and circumferential) constitute an interesting parameter in the assessment of aortic dilatation [11].

A detailed analysis of WSS permitted us to use a 3D representation of axial and circumferential WSS maps along the AAo, showing asymmetrical patterns that may contribute to structural changes in the aortic wall (elastin and metalloproteinases) related to aortic dilatation [8]. Furthermore, the presence of eccentric but uniform flow along the anterior AAo in the RL-phenotype determines that the axial component of WSS is greater in this subgroup of patients; however, the eccentric but helical flow in the RN-phenotype determines that the circumferential component of WSS is greater in this subgroup. This variation in WSS components may also influence the aortic morphotype. Thus, patients with a greater axial component exhibit more dilatation at the aortic root; however, greater circumferential WSS is associated with dilatation in the AAo.

Despite the correlations found here and elsewhere, the causative role of the observed flow disturbances need to be assessed in longitudinal studies. In particular, it has been mainly discussed in root only dilatation in BAV, which is thought to be a predominantly genetic form of BAV disease [9]. Our data confirmed the previously found association between root (only) dilatation and male sex [2].

The association of male sex, normalized displacement, and axial WSS in the proximal aorta discriminated the root-morphotype with an AUC of 0.91. However, the combination of an RN-phenotype, circumferential WSS and SFRR discriminated the ascending-morphotype with an AUC of 0.89. Thus, we believe these parameters should be considered in the evaluation of BAV beyond aortic diameters. This additional information could identify patients at higher risk of aortopathy that may require a closer follow-up.

### Limitations

The prospective nature of our study determined the inclusion of more patients with the RL-phenotype than RN-; however, our cohort reflects the distribution of fusion phenotypes in the general BAV population.

Healthy subjects were recruited to match BAV patients for age and aortic diameters. Although aortic diameters were slightly larger in BAV, these differences were not statistically significant.

Owing to the limited spatial and temporal resolution of 4D-flow, WSS is known to be underestimated [25, 34, 35]. However, all acquisitions were made with the same imaging parameters and analyzed with the same methodology and previous work highlighted that regions of high/low WSS are matched despite different spatial and temporal resolutions [35]. Additionally, manual segmentation causes intra- and inter-observer variability. Nevertheless, the robustness of WSS measurements employed in this study and their reproducibility has been previously demonstrated [36].

WSS estimation was limited to 8 slices in the AAo at peak systole. Thus, very localized regions of altered WSS may have been lost and temporal variations were not assessed. The use of a volumetric WSS method [37, 38], would allow a more detailed analysis.

Despite flow variables are likely to vary during the ejection phase, our measurements of jet angle, flow displacement, IRF and WSS were performed only at peak systole. Moreover, these measurements were performed averaging the results obtained at four successive time instants to reduce noise. Despite this approach has been used by several authors [7, 11, 13], and have proved high reproducibility [38], it may imply the loss of possible information contained in other systolic phases.

We conducted a cross-sectional study to evaluate the impact of flow dynamics in aortic dilatation in BAV. However, the real influence of these parameters on the concurrence of aortic dilatation needs to be determined in further longitudinal studies.

## Conclusions

BAV patients present altered flow patterns that vary depending on their valvular phenotype. RL-BAV patients present an anterior distribution, whereas, RN-BAV present a predominant posterior outflow jet at the sinotubular junction that shifts to anterior or right anterior in mid and distal AAo. This flow distribution induces an increase in the WSS_ax avg_ in the anterior aortic wall in RL-BAV patients, while, an increase in the in-plane rotational flow and WSS_circ avg_ in the mid and distal AAo in RN-BAV patients. These results may explain different AAo dilatation morphotypes in the BAV population. Thus, in addition to aortic diameters, the assessment of the different WSS components (axial and circumferential) and derived flow parameters may contribute to identify more completely, and precisely which patients have a higher risk of aortic dilatation. The follow-up of our series will permit validation of our results.

## Additional files

Additional file 1: Systolic flow in a RL-BAV patient. Systolic flow in a RL-BAV patient, showing a jet toward the anterior wall of the ascending aorta. (AVI 4858 kb)

Additional file 2: Systolic flow in a RN-BAV patient. Systolic flow in a RN-BAV patient, showing a posterior outflow jet that shifts anteriorly in the mid and distal ascending aorta. (AVI 3996 kb)

Additional file 3: Systolic flow in a healthy volunteer. Systolic flow in a healthy volunteer, showing a non-eccentric and laminar flow. (AVI 4694 kb)

## Abbreviations

4D-flow: Time-resolved three-dimensional phase-contrast magnetic resonance imaging
AAo: Ascending aorta
BAV: Bicuspid aortic valve
bSSFP: Balanced steady state free precession
CMR: Cardiovascular magnetic resonance
ECG: Electrocardiogram
FOV: Field of view
IRF: In-plane rotational flow
PC: Phase contrast
RL-BAV: Bicuspid aortic valve with fusion of the right and left coronary cusps
RN-BAV: Bicuspid aortic valve with fusion of the right coronary cusp and the non-coronary cusp
ROC: Region of interest
SFRR: Systolic flow reversal ratio
VENC: Velocity encoding
WSS: Wall shear stress
WSS_ax avg_: Circumferentially-averaged axial WSS
WSS_circ avg_: Circumferentially-averaged circumferential WSS
WSS_mag avg_: Circumferentially-averaged magnitude WSS
